# Outcome of Vacuum-Assisted Closure Technique in Skin Grafting Over Anatomically Challenging Areas

**DOI:** 10.7759/cureus.109264

**Published:** 2026-05-20

**Authors:** Md. Bayzid Bostamy, Aminur Rahid, Abid Azad, Masfik Ahmad, Lailun Nahar, Muhammad Noazesh Khan

**Affiliations:** 1 Department of Burn and Plastic Surgery, Shaheed Suhrawardy Medical College Hospital, Dhaka, BGD; 2 Department of Burn and Plastic Surgery, National Institute of Burn and Plastic Surgery, Dhaka, BGD; 3 Department of Burn and Plastic Surgery, TMSS Medical College, Bogura, BGD; 4 Department of Burn and Plastic Surgery, Dhaka Medical College Hospital, Dhaka, BGD

**Keywords:** bangladesh, graft survival, negative pressure wound therapy, skin transplantation, wound healing

## Abstract

Background

Vacuum-assisted closure (VAC) therapy is a well-established method that uses negative pressure to promote wound healing and enhance skin graft adherence. While globally accepted, outcomes following its use in anatomically challenging wound sites within the Bangladeshi population remain underexplored. This study aimed to assess graft take and postoperative complications following VAC-assisted skin grafting in anatomically challenging wound sites.

Methods

A prospective observational study was conducted at the National Institute of Burn and Plastic Surgery, Dhaka, from September 2020 to August 2021, involving 50 patients undergoing split-thickness skin grafting over difficult anatomical regions. VAC therapy was applied postoperatively at -90 to -125 mmHg for five days. Graft take was assessed on the 5th and 14th postoperative days and categorized as good (<10% loss), satisfactory (10%-25% loss), or poor (>25% loss). Postoperative complications were recorded. Statistical analyses included the chi-square test, Fisher’s exact test, and Pearson’s correlation, with p<0.05 considered significant.

Results

Complete (100%) graft take was achieved in 31 (62%) patients, while 41 (82%) demonstrated good overall graft outcomes. Complete graft take was more frequently observed in well-vascularized wound beds than in less vascularized wound beds (25/32 vs. 6/18, p=0.005). No significant correlation was observed between wound size and graft loss (r=0.0418, p=0.773). Younger patients (2-19 years) showed significantly better graft outcomes, whereas poor outcomes were confined to patients aged ≥40 years (p<0.001). Postoperative complications were minimal and transient.

Conclusion

VAC-assisted dressing was associated with favorable graft take and few short-term postoperative complications in anatomically challenging wound sites. However, due to the observational design, small sample size, absence of formal power analysis, and lack of a standard dressing control group, these findings should be interpreted cautiously and cannot establish relative efficacy compared with conventional dressing methods.

## Introduction

A skin graft is a procedure in which skin of variable thickness is harvested from a donor site and transplanted to cover a wound or tissue defect [1]. The long-term success of a skin graft depends on graft adherence and integration, which usually begins within 24 to 72 hours after grafting and is influenced by several local and systemic factors [2]. Immediate adherence of the graft to the recipient bed is critically important to prevent shearing movement. Seroma at the recipient site and wound infection are additional variables that may also prevent graft take at that point [3]. Successful grafting depends on proper wound bed preparation, careful patient selection, precise surgical technique, and close postoperative follow-up [4].

Clinical studies have demonstrated that Vacuum-assisted closure (VAC) or negative pressure wound therapy can improve split-thickness skin graft stabilization and graft take, particularly in irregular, mobile, or anatomically difficult wound sites [5,6]. By securing the graft uniformly to the wound bed, VAC therapy may also reduce the need for prolonged immobilization in difficult anatomical areas [7].

Morykwas et al. first introduced the VAC in 1997 as a non-pharmacologic, non-surgical method of wound healing [8]. Wound healing relies on creating an environment that minimizes inflammation and infection. VAC is a successful wound care method over the past two decades that reduces the need for amputations by using negative pressure to promote healing [9]. Debridement, controlled hemostasis, and the application of sterile foam dressings are all part of the procedure. A fenestrated connecting tube is attached to a vacuum pump that provides continuous or intermittent negative suction pressure, usually ranging from 50 to 125 mmHg. This therapy promotes primary wound closure by stabilizing the wound, lowering edema and bacterial load, enhancing tissue perfusion, and stimulating granulation and angiogenesis. It may reduce hospital stay and overall wound-care costs compared with conventional dressings [10]. It has become a major alternative method for graft fixation [5].

Although the technique is gaining popularity worldwide, very few studies have evaluated the outcome of VAC therapy in skin grafting in similar low-resource healthcare settings. Therefore, this study aimed to describe short-term graft take and postoperative complications following VAC-assisted split-thickness skin grafting (STSG) over anatomically challenging areas.

## Materials and methods

This prospective observational study was conducted from September 2020 to August 2021 at the National Institute of Burn and Plastic Surgery, Dhaka. A total of 50 patients with soft tissue defects in various body regions requiring STSG were enrolled through purposive sampling.

No formal a priori sample size calculation or power analysis was performed before patient enrollment. The sample size was determined pragmatically based on the number of eligible patients who underwent STSG with postoperative vacuum-assisted closure therapy during the study period. Therefore, this study should be interpreted as an observational preliminary study rather than a powered comparative efficacy trial.

Anatomically challenging wound sites were defined as areas with high mobility, contour irregularity, difficulty in immobilization, or relatively compromised graft adherence, including joints, axilla, groin, neck, perineum, and other irregular body surfaces. Patients with wounds located over these regions, or with irregular or less vascularized wound beds, were included in the study. Patients with grossly contaminated wounds and major uncontrolled comorbidities such as uncontrolled diabetes mellitus, psychiatric disorders, or polytrauma were excluded.

Routine preoperative assessments included complete blood count, erythrocyte sedimentation rate, random blood sugar, serum creatinine, hepatitis B surface antigen, and anti-hepatitis C virus screening. Wound size was measured in cm² using a measuring tape, and infection status was assessed by wound swab culture and sensitivity testing.

During surgery, wounds were debrided, and hemostasis was achieved using vasoconstrictors and diathermy. STSGs were harvested using a Humby knife, meshed at a 3:1 ratio when necessary, and secured with staples or sutures. The VAC dressing consisted of polyurethane foam covered with Opsite adhesive film and connected to a bedside vacuum pump. A continuous negative pressure ranging from -90 to -125 mmHg was applied for five days postoperatively.

The pressure level was selected according to wound size, anatomical location, graft stability, and patient tolerance. A lower pressure setting, approximately -90 mmHg, was generally used for smaller wounds, relatively stable grafts, less mobile anatomical sites, or when patient discomfort occurred. Higher pressure settings, up to -125 mmHg, were used for larger wounds, highly mobile areas such as joints, irregular contour regions, or grafts judged intraoperatively to require stronger fixation and more uniform suction. Pressure selection was based on the operating surgeon’s clinical judgment rather than a formal written protocolized pressure-selection algorithm. In some large wounds, an additional 18-G feeding tube was used to facilitate uniform distribution of negative pressure.

No additional immobilization techniques, such as splinting or plaster, were used, and early mobilization was encouraged from the first postoperative day. Graft assessment was performed on the 5th and 14th postoperative days. Graft take percentage and postoperative complications, including seroma, hematoma, infection, and shearing, were evaluated clinically. Graft take was assessed by the operating surgical team during postoperative follow-up. Blinded assessment was not performed because the VAC dressing and surgical details were known to the treating team. The percentage of graft loss was estimated by clinical visual inspection of the grafted area. Graft take was categorized as good when graft loss was <10%, satisfactory when graft loss was 10%-25%, and poor when graft loss was >25%.

Data were analyzed using IBM SPSS Statistics version 22.0 (IBM Corp., Armonk, NY). Categorical variables were summarized as frequencies and percentages, while continuous variables were expressed as mean ± standard deviation. Associations between categorical variables were assessed using the Pearson’s chi-square test and Fisher’s exact test, where applicable. Correlation between continuous variables was evaluated using Pearson’s correlation. A p-value of less than 0.05 was considered statistically significant.

Ethical approval

Ethical approval was obtained from the institutional ethics committee before the commencement of the study (memo no: SHNIBPS/ECC/1835). Written informed consent was obtained from all participants. All procedures were conducted in accordance with the Declaration of Helsinki.

## Results

In this study, the majority of patients were male, 39 (78%), and predominantly younger in age, with 19 (38%) aged 2-19 years and 18 (36%) aged 20-39 years (Table 1).

**Table 1:** Baseline demographics of the study participants (n=50)

Characteristic	Frequency (percentage)
Age group	
2-19 years	19 (38%)
20-39 years	18 (36%)
≥40 years	13 (26%)
Gender	
Female	11 (22%)
Male	39 (78%)

Among the study participants, the majority of wounds were of moderate size, with 18 (36%) measuring 1-200 cm² and 16 (32%) measuring 201-400 cm². Nearly half of the wounds, 23 (46%), were located over joint areas, while 21 (42%) involved contour regions. Irregular wound beds were identified in 19 (38%) cases, and 18 (36%) had less vascularized wound beds. Comorbidities were present in a small proportion of patients, 6 (12%), predominantly diabetes (Table 2).

**Table 2:** Clinical characteristics of the wounds

Characteristic	Frequency (percentage)
Size of wound (cm^2^)	
1-200	18 (36%)
201-400	16 (32%)
401-600	8 (16%)
601-800	7 (14%)
800	1 (2.0%)
Wound over the joint	23 (46%)
Contour region	21 (42%)
Wound bed	
Irregular	19 (38%)
Regular	31 (62%)
Wound bed vascularity	
Less vascularized	18 (36%)
Well vascularized	32 (64%)
Comorbidities	
Diabetic	5 (10%)
Malignancy	1 (2.0%)
No	44 (88%)

**Figure 1:**
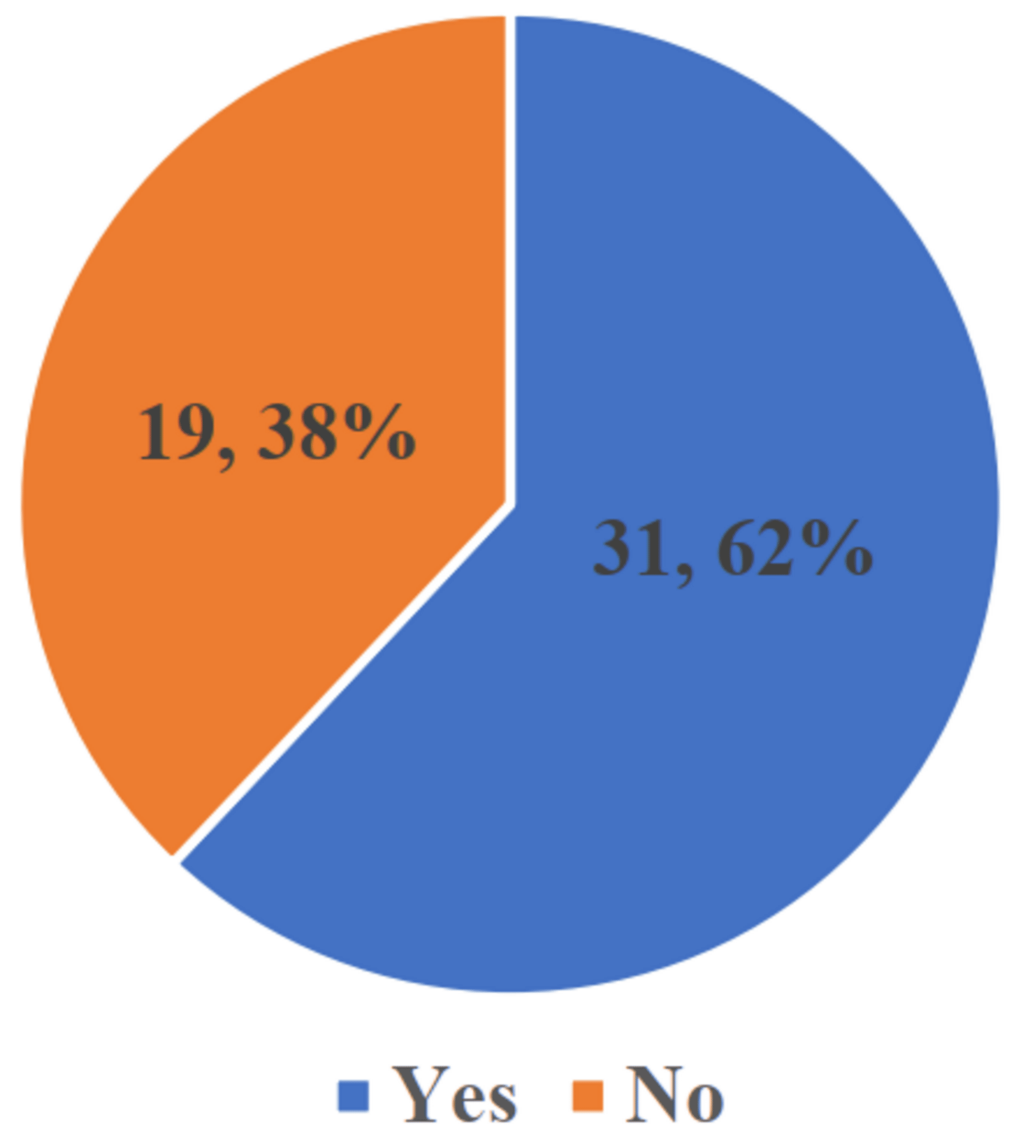
Status of 100% graft taking

In this study, 31 (62%) patients achieved complete graft take after VAC-assisted graft fixation (Figure 1).

**Figure 2:**
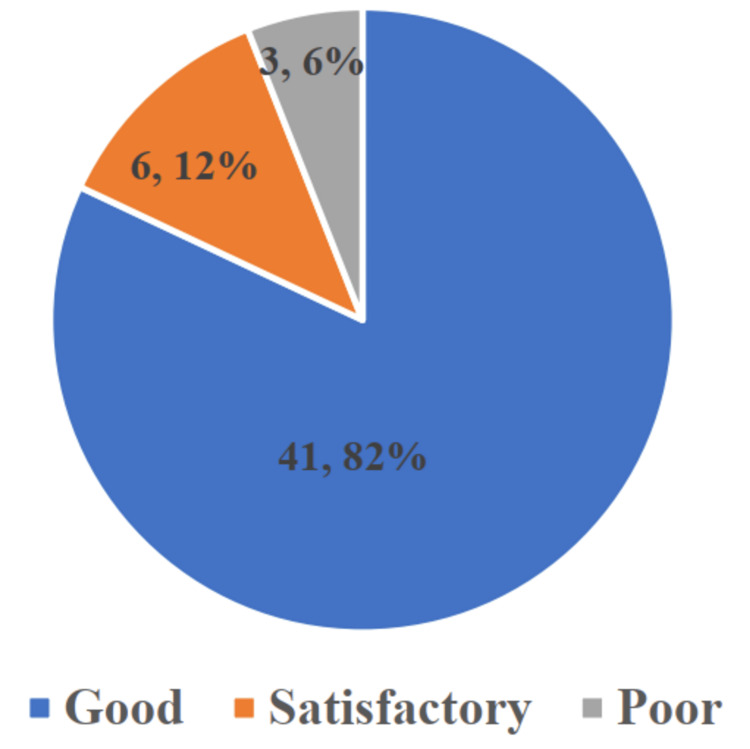
Satisfactory level of graft takes

Based on categorical assessment, 41 (82%) patients had good graft outcomes, while 6 (12%) had satisfactory outcomes and 3 (6%) had poor outcomes (Figure 2).

Table 3 showed that complete (100%) graft take was more common in wounds with well-vascularized beds, observed in 25 (81%) patients, compared to 6 (19%) patients with less vascularized beds, demonstrating a statistically significant association (χ²=7.80, df=1, p=0.005). Other factors, including wound location over joints, contour regions, and irregular wound beds, were not significantly associated with graft take (p>0.05).

**Table 3:** Association between 100% graft take and clinical factors

Characteristic	100% graft take		χ²	p-value
	No (N=19)	Yes (N=31)		
Wound over the joint	9 (47%)	14 (45%)	0.84	0.9
Contour region	10 (53%)	11 (35%)	1.42	0.2
Wound bed				
Irregular	9 (47%)	10 (32%)	1.14	0.3
Regular	10 (53%)	21 (68%)		
Wound bed vascularity				
Less vascularized	11 (58%)	6 (19%)	7.8	0.005
Well vascularized	8 (42%)	25 (81%)		

Table 4 showed that postoperative complications were minimal and transient, with seroma in 4 (8%), shearing in 2 (4%), hematoma in 1 (2%), and infection in 1 (2%) patients observed only at the first follow-up, all of which resolved by the second follow-up.

**Table 4:** Postoperative complications POD, postoperative day.

Characteristic	5^th^ POD (1^st^ follow-up)	14^th^ POD (2^nd^ follow-up)
	n (%)	n (%)
Seroma	4 (8.0)	0 (0.0)
Hematoma	1 (2.0)	0 (0.0)
Shearing	2 (4.0)	0 (0.0)
Infection (Pseudomonas)	1 (2.0)	0 (0.0)

This study found no significant correlation between wound size and graft loss (r=0.0418, p=0.7731) (Table 5).

**Table 5:** Correlations between wound size and graft loss (n=50)

Characteristic	r-value	p-value
Size of wound (cm²)	0.0418	0.773
Graft loss (%)		

A significant association was observed between age group and graft outcome (p<0.001). Good outcomes were observed in 17/19 (89.5%) patients aged 2-19 years, 15/18 (83.3%) aged 20-39 years, and 9/13 (69.2%) aged ≥40 years, while all poor outcomes occurred in patients aged ≥40 years (Table 6).

**Table 6:** Association of graft outcome with age groups

Characteristic	Good (N=41)	Satisfactory (N=6)	Poor (N=3)	p-value
Age group				
2-19 years	17 (41%)	0 (0%)	0 (0%)	<0.001
20-39 years	15 (37%)	0 (0%)	0 (0%)	
≥40 years	9 (22%)	6 (100%)	3 (100%)	

## Discussion

The present study found that 31 (62%) patients achieved complete graft take and 41 (82%) had good graft outcomes following VAC-assisted graft fixation. These findings indicate favorable short-term graft outcomes in anatomically challenging areas. However, because this study did not include a standard dressing comparison group, the results should not be interpreted as evidence of superiority or relative efficacy of VAC therapy over conventional dressing methods. Cao et al. reported a higher graft take rate with negative pressure wound therapy than with traditional dressings, supporting the beneficial role of negative pressure therapy in improving skin graft outcomes [6].

The role of wound bed vascularity in graft success was clearly revealed in this study. Patients with well-vascularized wound beds demonstrated significantly higher rates of 100% graft take (81%) than those with poorly vascularized beds (19%) (p=0.005). This finding may reflect the importance of adequate wound bed vascularity for graft survival. Although VAC therapy may help maintain graft contact, remove exudate, and support a favorable wound environment, the present study cannot independently determine the mechanistic contribution of VAC therapy to improved perfusion or granulation. Previous evidence also suggests that negative pressure wound therapy can create a favorable wound environment by improving perfusion, reducing edema, and supporting granulation tissue formation, which may enhance graft take [11].

Although wound characteristics may influence graft take [12], our findings did not show any significant correlation between wound size and graft loss. This finding is consistent with evidence suggesting that graft outcome may depend more on wound bed condition and postoperative care than on wound size alone [13]. This finding may be related to uniform surgical technique, standardized postoperative care, or consistent application of VAC-assisted fixation across varying wound sizes, minimizing the potential impact of wound dimensions on graft outcome.

A significant association was observed between age group and graft outcome in this sample, with good outcomes more frequently observed among younger patients and poor outcomes among patients aged ≥40 years. Baharestani et al. also reported favorable outcomes with VAC therapy in pediatric wounds, with high graft take and minimal infection [14]. This may be attributed to better regenerative capacity and fewer comorbidities in younger individuals, supporting patient selection as a consideration in surgical planning. Conversely, poor graft outcomes were confined to the ≥40 years age group, which may be due to age-related delayed wound healing, reduced skin elasticity, compromised vascular supply, and comorbid conditions such as diabetes or hypertension. Another study conducted on 60 patients (mean age ~53 years) revealed that comorbidities and age-related factors such as poor nutritional status and elevated inflammatory markers were significantly associated with delayed healing and complications, suggesting poorer outcomes in older patients receiving VAC therapy [15].

Postoperative complications were minimal and self-limiting, with seroma (8%), shearing (4%), hematoma (2%), and infection (2%) observed only at the initial follow-up and fully resolving by day 14. In a prospective study, 19 out of 20 patients treated with VAC therapy had successful wound healing, with minimal postoperative complications reported. The study emphasized VAC as a reliable, safe method for chronic wound management with favorable graft take and reduced hospital stay [16]. Previous meta-analytic evidence has suggested that negative pressure wound therapy may improve graft-related outcomes and reduce complications in selected settings. However, the present study can only describe the observed short-term complication profile because it lacked a comparator group [17]. The minimal and self-limiting complications may be due to VAC therapy’s ability to enhance wound healing, reduce bacterial load, and maintain a controlled environment. These mechanisms may partly explain the low frequency of short-term postoperative complications observed in this study.

Despite its useful observations, this study had several limitations. The sample size was small, no formal sample size calculation or power analysis was performed, and the single-center purposive sampling design may limit generalizability and introduce selection bias. As no conventional dressing control group was included, the relative efficacy of VAC therapy compared with standard graft fixation methods could not be determined. Graft take was assessed clinically by the treating surgical team without a blinded independent assessment, so observer bias and subjective estimation could not be excluded. In addition, pressure selection was based on surgeon judgment rather than a formal protocolized algorithm, which may have introduced treatment heterogeneity. The short follow-up period also limited evaluation of long-term graft durability, scar quality, functional outcomes, and patient-reported quality of life. Therefore, larger multicenter comparative studies with adequate power, standardized pressure criteria, blinded outcome assessment, longer follow-up, and cost-effectiveness analysis are recommended.

## Conclusions

In this study, VAC-assisted dressing was associated with favorable short-term graft take and few transient postoperative complications among patients undergoing split-thickness skin grafting over anatomically challenging areas. Complete graft take was more commonly observed in well-vascularized wound beds, while poorer outcomes were seen mainly among older patients in this sample. This study cannot determine whether VAC therapy is superior to conventional dressing methods. Larger, adequately powered comparative studies with standardized pressure-selection criteria and blinded outcome assessment are needed to confirm these findings.
